# Identification of Urinary Activin A as a Novel Biomarker Reflecting the Severity of Acute Kidney Injury

**DOI:** 10.1038/s41598-018-23564-3

**Published:** 2018-03-26

**Authors:** Shunsuke Takahashi, Masao Nakasatomi, Yoshinori Takei, Hidekazu Ikeuchi, Toru Sakairi, Yoriaki Kaneko, Keiju Hiromura, Yoshihisa Nojima, Akito Maeshima

**Affiliations:** Department of Nephrology and Rheumatology, Gunma University Graduate School of Medicine, Maebashi, Japan

## Abstract

Acute kidney injury (AKI) is a common but complex condition that is associated with increased morbidity and mortality. In the present study, we examined whether urinary activin A, a member of the TGF-beta superfamily, is present in mice with ischemia-reperfusion injury and in humans with AKI, as well as its potential as a biomarker for AKI. Expression of activin A was markedly increased in ischemic mouse kidneys. *In situ* hybridization demonstrated that activin mRNA was expressed in tubular cells of ischemic kidneys but not of normal kidneys. Immunoreactive activin A, which was absent in normal kidneys, was detected in the cytoplasm of proximal tubular cells in ischemic kidneys. Activin A was undetectable in the urine of normal mice. In contrast, activin A was significantly increased in the urine of ischemic mice at 3 h after reperfusion. Urinary activin A levels increased according to the period of ischemia. In humans, urinary activin A was almost undetectable in healthy volunteers and in patients with pre-renal AKI, but was significantly increased in patients with renal AKI. There was no significant correlation between urinary activin A and serum activin A. Collectively, urinary activin A might be a useful biomarker reflecting the severity of AKI.

## Introduction

Acute kidney injury (AKI) represents a very important and potentially devastating disorder in clinical medicine. The incidence of AKI is increasing to epidemic proportions. AKI is associated with prolonged hospital stay, increased healthcare costs and high mortality in critically ill patients. Unfortunately, serum creatinine is an unreliable indicator during acute changes in kidney function, and does not accurately reflect kidney function until a steady state has been reached. The lack of early biomarkers for AKI has impaired our ability to intervene in a timely manner^1^.

Recently, several new AKI biomarkers have been developed to facilitate early detection, differential diagnosis, and prognosis, and which include neutrophil gelatinase-associated lipocalin (NGAL)^2–5^, kidney injury molecule 1 (KIM-1)^6–8^, interleukin 18 (IL-18)^9,10^ and liver-type fatty acid-binding protein (L-FABP)^11–13^.

Activin is a multifunctional cytokine belonging to the TGF-β superfamily that regulates the growth and differentiation of cells in various organs^14^. Its action is modulated by an endogenous activin antagonist, follistatin^15^. Activin signals are mediated by two types of cell surface serine/threonine receptors. Activin first binds to the type II receptor (ActRII or ActRIIB), which leads to recruitment and phosphorylation of the type I receptor (ActRI or ActRIB) and formation of receptor complexes. The activated type I receptor then phosphorylates Smad proteins, which are subsequently translocated to the nucleus and regulate target gene expression^16^. Activin A is expressed in the developing stage of the kidney^17^. Organ culture experiments demonstrated that activin A inhibits branching morphogenesis of ureteric buds^18–20^ as well as ureteric bud budding from the Wolffian duct^21^. In an *in vitro* tubulogenesis model using Madin-Darby canine kidney (MCDK) cells, activin A tonically inhibited branching tubulogenesis. On the other hand, blockade of endogenous activin A action by follistatin induced branching tubulogenesis^22^, suggesting that activin A negatively regulates tubulogenesis during kidney organogenesis^23,24^.

Previously, we demonstrated that activin A expression was significantly increased in tubular cells of the kidney after renal ischemia in rats. Furthermore, intravenously administered follistatin, which binds to activins and block their actions, improved renal dysfunction and histological changes after renal ischemia^25,26^. We also demonstrated that activin A acts as an autocrine inhibitor of cell growth as well as an inducer of apoptosis in cultured proximal tubular cells^27^, suggesting that activin A negatively regulates tubular repair of the kidney after AKI.

In the present study, we demonstrated the presence of activin A in the urine of mice with renal ischemia-reperfusion injury. Activin A expression was markedly upregulated in proximal tubular cells of ischemic kidneys. Urinary activin A level was correlated with the degree of tubular damage. Urinary activin A was also significantly increased in patients with renal AKI, but not with pre-renal AKI, suggesting that urinary activin A might serve as a sensitive biomarker reflecting AKI severity.

## Results

### Expression of βA subunit for Activin A in the Kidneys of Mice after Renal Ischemia

**Figure 1:**
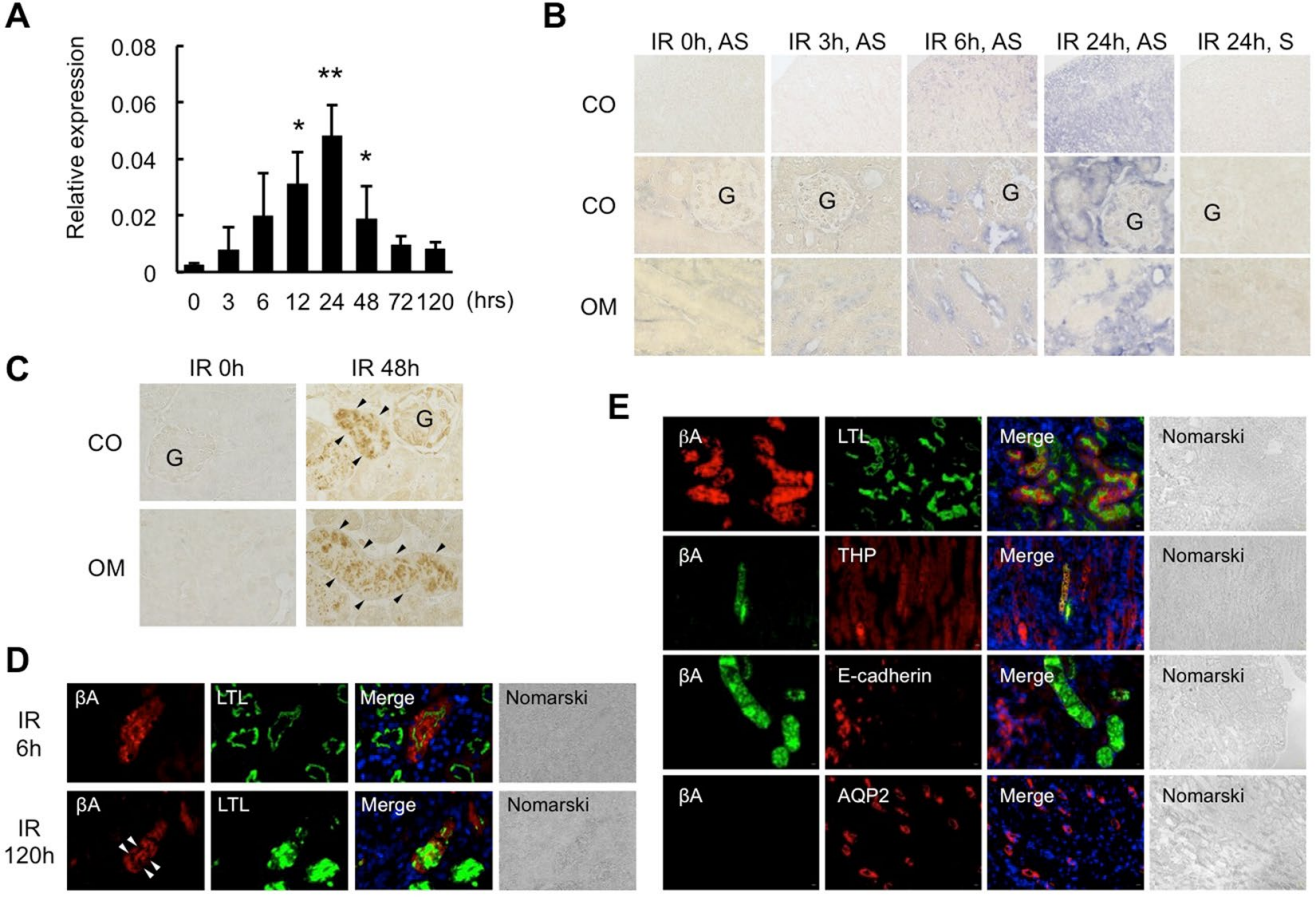
Expression of βA Subunit for Activin in the Kidneys after Renal Ischemia. (**A**) Expression of βA subunit for activin in the kidneys of mice after renal ischemia for 25 min was examined by real-time PCR. Values (relative expression ratio to GAPDH) are means ± S.E. (n = 5–8). *p < 0.05, **p < 0.01 vs. 0 hr. (**B**) Localization of βA subunit mRNA in the kidneys after renal ischemia was examined by *in situ* hybridization. Cortex: CO, Outer medulla: OM. G: glomerulus. Hybridization signals are shown as blue color. AS, anti-sense probe, S, sense probe. Magnification: ×100 (upper panels) and ×1000 (middle, lower panels). (**C**) Localization of activin A in the kidneys after renal ischemia was examined by immunostaining. Activin A (brown). Arrowheads indicate activin-positive renal tubules. Cortex: CO, Outer medulla: OM. Magnification: ×1000. (**D**) Double-staining of activin A with LTL in the ischemic kidneys at 6 h (upper panels) and 120 h (lower panels) after reperfusion. Activin A (red), LTL (green), DAPI (blue). Magnification, ×1000. Arrows indicate activin A-positive casts in the lumen of renal tubules. (**E**) Double-staining of activin A with several nephron markers in the kidneys after renal ischemia. LTL (green), THP (red), E-cadherin (red) and aquaporin 2 (AQP2; red), and DAPI (blue). Magnification, ×400.

To examine whether activin A is expressed in ischemic kidneys, we first analyzed the mRNA expression of βA subunit for activin A in the kidneys of mice after renal ischemia for 25 min using real-time PCR (Fig. 1A). The expression of βA subunit mRNA, which was almost undetectable in normal kidneys, was significantly increased in ischemic kidneys and peaked at 24 hr after reperfusion. Next, we examined the localization of βA subunit mRNA in normal and ischemic kidneys by *in situ* hybridization (Fig. 1B). Hybridization signals were not observed in normal kidneys and ischemic kidneys at 3 hr after reperfusion. In contrast, strong hybridization signals for βA subunit mRNA was observed, mainly in tubular cells of the outer medulla of the ischemic kidneys, at 6 hr after reperfusion and thereafter. There were no hybridization signals in the glomeruli of the ischemic kidneys. A control experiment using a sense probe showed no hybridization signal.

Localization of activin A was also examined by immunostaining. The area of cortex containing glomeruli, outer medulla, and inner medulla were identified by the anatomical structures of the kidney specimens. Activin A was detected in tubular cells of the cortex (Fig. 1C, right upper panel) and outer medulla (Fig. 1C, right lower panel) of ischemic kidneys, but not in normal kidneys (Fig. 1C, left panels). Activin A was localized in the cytoplasm of LTL-positive proximal tubular cells at 6 hr after reperfusion (Fig. 1D, upper panels). At the later phase of AKI (120 hr after reperfusion), there were many activin A-positive casts in the lumen of renal tubules (Fig. 1D, lower panels). Activin A was also detected in the THP-positive ascending limb of loop of Henle, but not in E-cadherin-positive distal tubular cells and AQP2-positive collecting ducts in ischemic kidneys at 48 hr after reperfusion (Fig. 1E).

**Figure 2:**
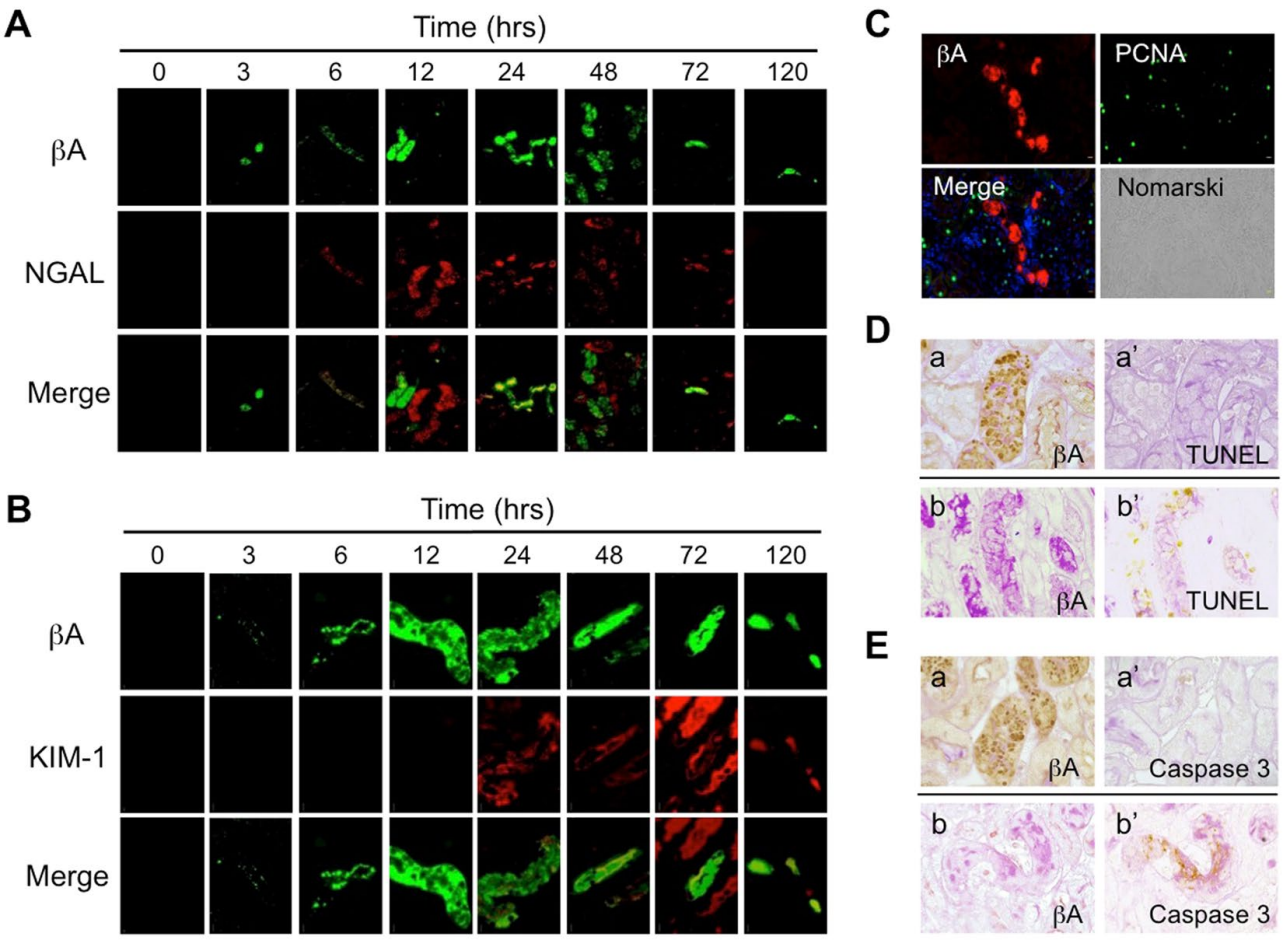
Localization of A subunit for Activin, NGAL, and KIM-1 in the Kidneys after Renal Ischemia. (**A**) Localization of βA subunit for activin and NGAL in the kidneys after renal ischemia for 25 min was examined by immunostaining. βA subunit (green), NGAL (red). Magnification: ×400. (**B**) Localization of βA subunit for activin and KIM-1 in the kidneys after renal ischemia was examined by immunostaining. βA subunit (green), KIM-1 (red). Magnification: ×1000. (**C**) Localization of βA subunit for activin and PCNA in the ischemic kidneys at 48 hr after reperfusion. βA subunit (red), PCNA (green), and DAPI (blue). Magnification: ×400. (**D**) Immunostaining of βA subunit for activin (a, b) and TUNEL staining (a’,b’) in the ischemic kidneys at 48 hr after reperfusion using serial sections (a-a’,b-b’).Positive signals (brown). PAS-positive brush border (red). Magnification: ×1000. (**E**) Immunostaining of βA subunit for activin (a,b) and caspase 3 (a’,b’) in the ischemic kidneys at 48 hr after reperfusion using serial sections (a-a’,b-b’). Positive signals (brown). PAS-positive brush border (red). Magnification: ×1000.

We further compared the localization of activin A and other AKI biomarkers, such as NGAL and KIM-1, in the kidney after renal ischemia for 25 min. NGAL was present in tubular cells of the ischemic kidneys from 6 to 72 hr after reperfusion. Activin A was partly co-localized with NGAL in the ischemic kidneys (Fig. 2A). KIM-1 was also present in tubular cells of the ischemic kidneys. Co-localization of activin A and KIM-1 was observed in the ischemic kidneys at 24 hr after reperfusion and thereafter (Fig. 2B).

To characterize activin A-producing cells, we examined the localization of PCNA-positive proliferating cells in the kidney after renal ischemia (Fig. 2C). Many PCNA-positive cells were observed in the ischemic kidneys at 48 hr after reperfusion, but none were co-localized with activin A. There were many apoptotic cells positive for TUNEL or active caspase-3 in the ischemic kidneys. However, activin A was not co-localized with TUNEL (Fig. 2D) or active caspase-3 (Fig. 2E). Activin A-positive renal tubules (Fig. 2D-a) were TUNEL-negative (Fig. 2D-a’). On the other hand, TUNEL-positive cells (Fig. 2D-b’) were activin A-negative (Fig. 2D-b). Similarly, activin A-positive renal tubules (Fig. 2E-a) were negative for active caspase 3 (Fig. 2E-a’) and active caspase 3-positive cells (Fig. 2E-b’) were activin A-negative (Fig. 2E-b). These results suggest that the regulation of activin A expression was not associated with cell proliferation or apoptosis during tubular regeneration after injury.

### Detection of Activin A in the Urine of Mice with Ischemia-Reperfusion Injury

**Figure 3:**
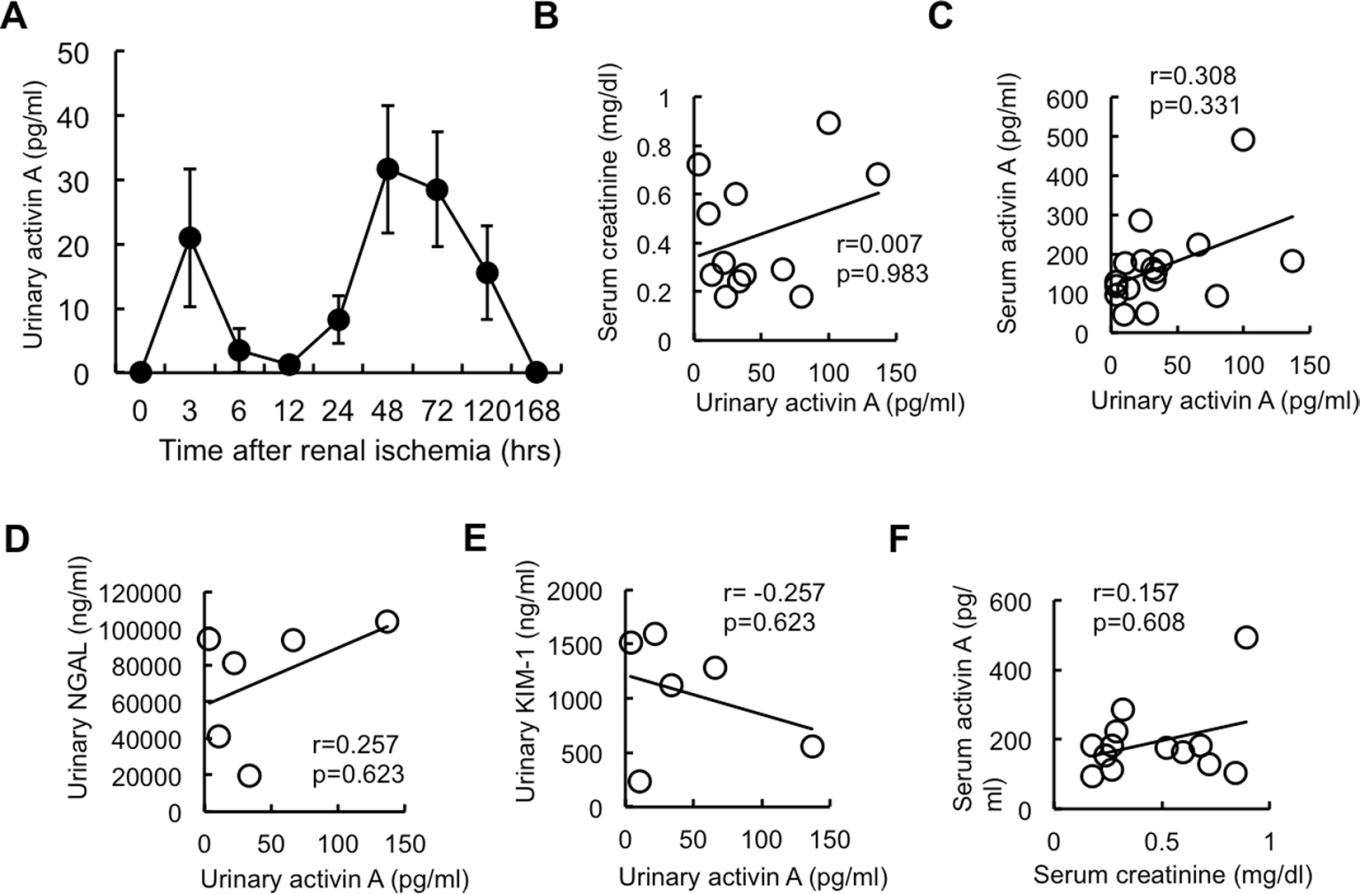
Urinary Activin A Level in Mice with Ischemia-reperfusion Injury. (**A**) Urinary activin A in mice with renal ischemia for 22 min was measured by ELISA. Values are means ± S.E. (n = 5–11). (**B**,**C**) Correlation between urinary activin A and serum creatinine (**B**) or serum activin A (**C**) at 48 hr after reperfusion. (**D**,**E**) Correlation between urinary activin A and urinary NGAL (**D**) or urinary KIM-1 (**E**) at 48 hr after reperfusion. (**F**) Correlation between serum creatinine and serum activin A at 48 hr after reperfusion.

We next examined whether urinary activin A was detectable in mice with AKI. Renal ischemia for 22 min was induced in mice and urine was collected for analysis at the indicated periods after reperfusion. Severe AKI with renal ischemia for more than 25 minutes occasionally induced anuria or oliguria. Therefore, we selected 22 minutes as ischemic period to collect adequate amount of urine from AKI model mice. Urinary activin A was measured by ELISA. Activin A was absent in the urine of normal mice; in contrast, activin A was detected in the urine of ischemic mice and a bimodal peak was observed at 3 and 48 hr after renal ischemia (Fig. 3A).

Next, we examined the correlation of urinary activin A with other parameters, such as serum creatinine (Fig. 3B), serum activin A (Fig. 3C), urinary NGAL (Fig. 3D), and urinary KIM-1 (Fig. 3E). Previous studies demonstrated that urinary Ngal increased at 12 hours after reperfusion and thereafter^5^. Urinary KIM-1 also increased after renal ischemic injury and peaked at 48 hours after reperfusion^8^. Similar to these data, urinary activin A increased and peaked at 48 hours after reperfusion. Therefore, we used urine samples collected at 48 hours after reperfusion to perform the correlation analysis. Urinary activin A was not correlated with any of these parameters (Fig. 3B–E). Moreover, there was no significant correlation between serum activin A and serum creatinine (Fig. 3F).

### Correlation of Urinary Activin A Level with Severity of AKI

**Figure 4:**
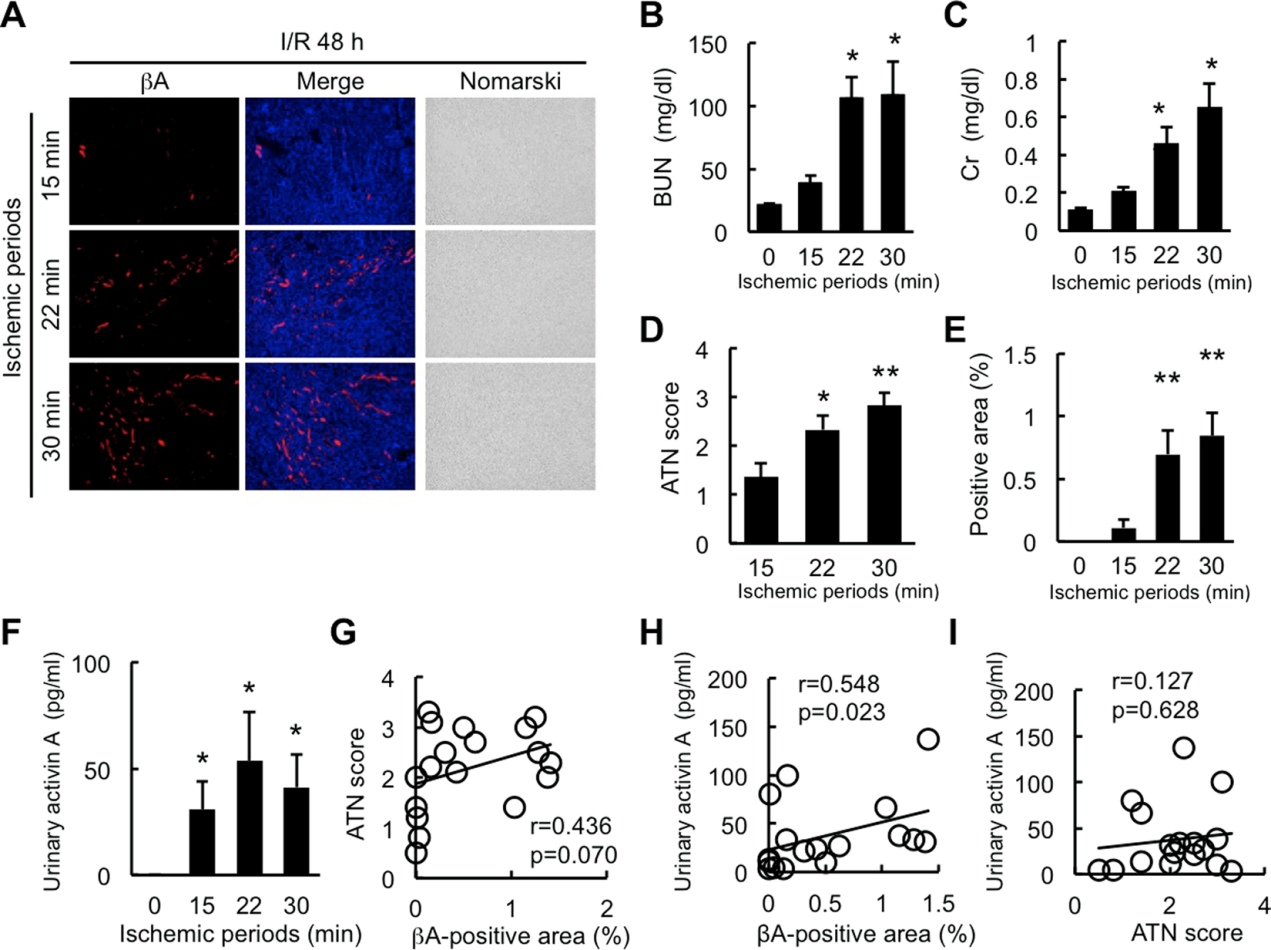
Correlation of Urinary Activin A with the Severity of Kidney Damage. (**A**) Localization of βA subunit for activin in the kidneys of mice with renal ischemia for 15, 22, or 30 min. The kidneys were removed for analysis at 48 hr after reperfusion. βA subunit for activin (red), DAPI (blue). Magnification: ×100. (**B**,**C**) BUN (**B**) and serum creatinine (**C**) in mice with renal ischemia at 48 hr after reperfusion. *p < 0.05, **p < 0.01 vs. 0 hr. (**D**) Semiquantitative analysis of the histologic changes induced by renal ischemia. ATN score was quantified as described in the Methods. *p < 0.05, **p < 0.01 vs. 15 min ischemia. (**E**) Quantitative analysis of positive area of βA subunit for activin. Five randomly selected fields of the kidneys were assessed at ×200 magnification. Activin A-positive area was measured using ImageJ software. Values are means ± S.E. (n = 6). **p < 0.01 vs. 15 min ischemia. (**F**) Urinary activin A in mice with renal ischemia for 15, 22, or 30 min. Urine was collected at 48 hr after reperfusion and urinary activin A was measured by ELISA. Values are means ± S.E. (n = 6). *p < 0.05 vs. 0 hr. (**G**,**H**) Correlation between activin A-positive area and ATN score (**G**) or urinary activin A (**H**) at 48 hr after reperfusion. (**I**) Correlation between ATN score and urinary activin A level at 48 hr after reperfusion.

We then examined the correlation of urinary activin A with the severity of kidney damage. Mild (15 min), moderate (22 min), and severe (30 min) renal ischemia was induced in C57BL/6j mice. Serum, urine and kidney tissues were collected at 48 hr after reperfusion. Immunostaining showed that activin A was detected in the ischemic kidneys regardless of ischemic periods (Fig. 4A). BUN, serum creatinine and ATN score significantly increased in mice with renal ischemia for 22 and 30 min, but not for 15 min (Fig. 4B–D). Quantitative analysis demonstrated that activin A-positive area significantly increased in the kidneys of mice with renal ischemia for 22 and 30 min, but not for 15 min (Fig. 4E). Urinary activin A level was significantly increased in mice with renal ischemia at 48 hr after reperfusion regardless of ischemic period (Fig. 4F).

We examined the correlation between ATN score, activin A-positive area, and urinary activin A. There was no significant correlation between ATN score and activin A-positive area (Fig. 4G). Urinary activin A was significantly correlated with activin A-positive area (Fig. 4H), but there was no correlation between urinary activin A and ATN score (Fig. 4I).

### Urinary Activin A Level in Patients with Acute Kidney Injury

**Figure 5:**
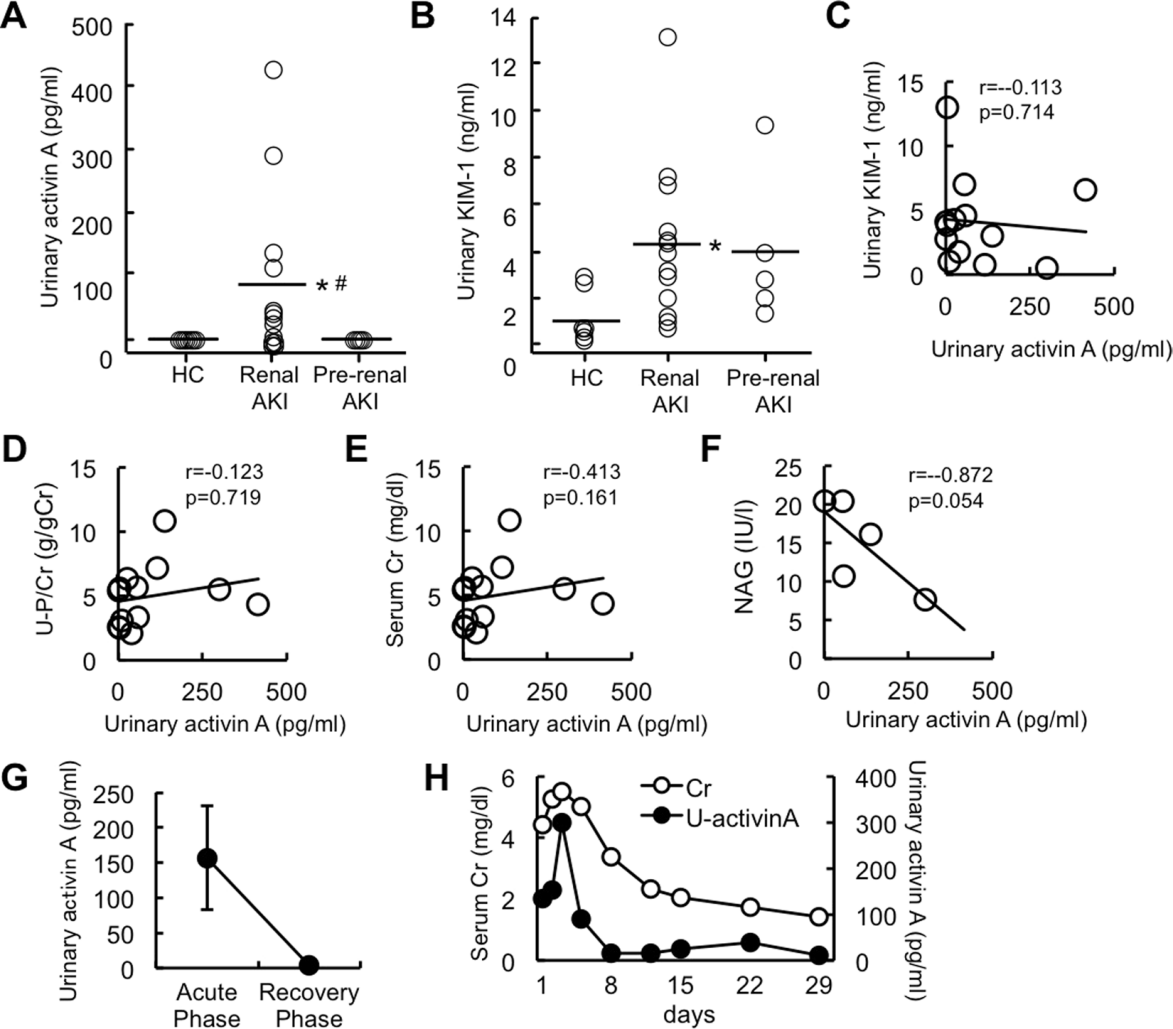
Urinary Activin A Level in Patients with Acute Kidney Injury. (**A**) Urinary activin A in healthy controls (n = 8), renal AKI patients (n = 13) and pre-renal AKI patients (n = 5). *p < 0.05 vs. healthy controls, ^#^p < 0.05 vs. pre-renal AKI patients. (**B**) Urinary KIM-1 in healthy controls (n = 7), renal AKI patients (n = 13) and pre-renal AKI patients (n = 5). *p < 0.05 vs. healthy controls. (**C**–**H**) Correlation between urinary activin A and urinary KIM-1 (**C**), urinary protein level (**D**), serum creatinine (**E**) or urinary NAG (**F**). (**G**) Urinary activin A level at the acute and recovery phases of AKI (n = 3). Data at the initial visit (acute phase) and before discharge (recovery phase) were shown. (**H**) Time course changes of urinary activin A and serum creatinine in patient with drug-induced AKI.

We also analyzed urinary activin A in eighteen patients with renal AKI and pre-renal AKI. The baseline characteristics of patients are shown in Table 1. Similar to the mouse AKI model described above, urinary activin A was significantly increased in renal AKI patients, but was not detected in healthy controls or in pre-renal AKI patients (Fig. 5A). Urinary KIM-1 also increased significantly in patients with renal AKI, but not with pre-renal AKI (Fig. 5B). At a cut-off value of 2.23 pg/ml, sensitivity was 0.846, specificity was 1.00, positive predictive value was 1.00 and negative predictive value was 0.800 for predicting renal AKI. We examined the correlation of urinary activin A with urinary KIM-1, urinary protein level, serum creatinine, and urinary NAG. None of these parameters were correlated with urinary activin A (Fig. 5C–F).

**Table 1:** Baseline Characteristics of AKI Patients.

	Total	Renal AKI	Pre-renal AKI	P-value (renal vs. pre-renal)
Number	18	13	5	
Age (years)	63.3 ± 3.46	63.7 ± 4.46	62.2 ± 6.30	0.849
Sex (M/F)	10/8	6/7	4/1	
U-Prot/U-Cr (g/gCr)	2.25 ± 0.55	2.91 ± 0.64	0.54 ± 0.18	0.003
sCr (mg/dl)	4.86 ± 0.50	4.96 ± 0.67	4.59 ± 0.61	0.688
BUN (mg/dl)	65.3 ± 6.22	59.8 ± 7.78	79.6 ± 7.08	0.083
Hb (g/dl)	9.31 ± 0.55	8.30 ± 0.43	11.9 ± 0.92	0.012
WBC (/μl)	5,233 ± 795.8	4,892 ± 880.0	6,120 ± 1,836	0.569
Plt (×10^4^)	14.0 ± 2.50	13.7 ± 3.13	14.6 ± 4.40	0.872
Na (mEq/L)	138 ± 1.24	137 ± 1.30	141 ± 2.68	0.192
K (mEq/L)	4.46 ± 0.23	4.40 ± 0.27	4.60 ± 0.46	0.729
Cl (mEq/L)	103 ± 1.27	102 ± 1.55	106 ± 1.60	0.077
NAG (IU/L)	19.3 ± 4.30	15.1 ± 2.56	26.7 ± 10.4	0.404

Age, sex, urinary protein level, serum creatinine, BUN, hemoglobin, WBC, platelet, sodium, potassium, chloride, and urinary NAG of patients with renal AKI (n = 13) (drug-induced 5, hypercalcemia 1, rhabdomyolysis 1, sepsis 1, granulomatosis with polyangiitis 1, HELLP syndrome 1, and cast nephropathy 3) and pre-renal AKI (n = 5) (dehydration) at the initial visit are shown. Data collected from patients with renal AKI (drug-induced 5, hypercalcemia 1, rhabdomyolysis 1, sepsis 1, granulomatosis with polyangitiis 1, HELLP syndrome 1, and cast nephropathy 3) and pre-renal AKI (dehydration 5) at the initial visit are shown.

A significant increase in urinary activin A was observed at the acute phase of AKI, but became almost undetectable at the recovery phase of AKI (Fig. 5G). In one case of drug-induced AKI, urinary activin A increased in parallel with serum creatinine and rapidly decreased before the normalization of serum creatinine level (Fig. 5H).

### Urinary Activin A Level in Volume Depletion Model Mice

**Figure 6:**
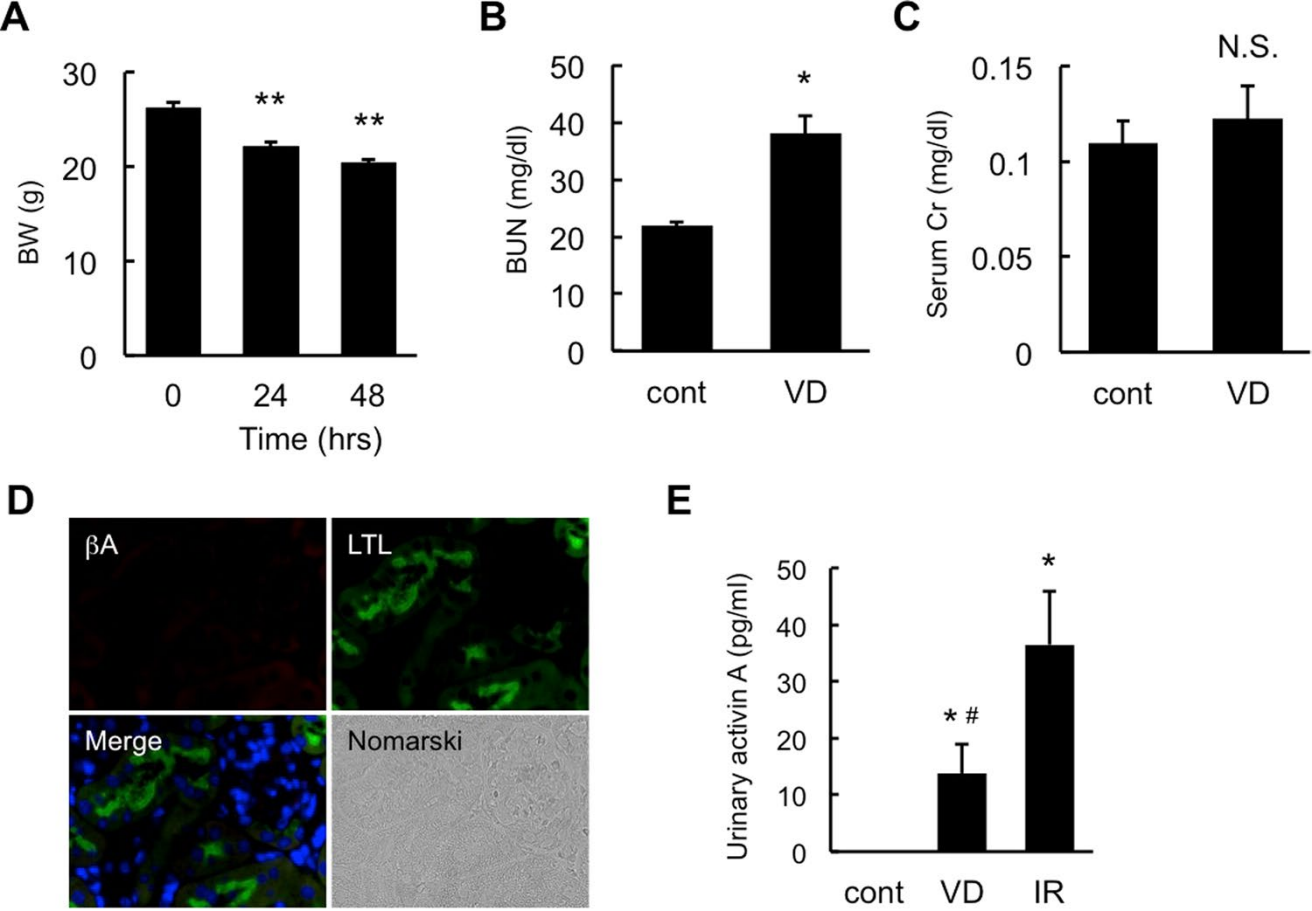
Urinary Activin A Level in Mice with Volume Depletion. (**A**) Changes of body weight in the volume depletion model. Simple volume depletion was induced in mice by water intake restriction to mimic pre-renal AKI. Values are mean ± S.E. **p < 0.01 vs. 0 hr. (**B**,**C**) Blood urea nitrogen (BUN) (**B**) and serum creatinine (**C**) in mice before and after 48 hr volume depletion. Values are mean ± S.E. *p < 0.05 vs. control (n = 6). N.S., not significant. (**D**) Localization of βA subunit for activin (red) and LTL (green) in the kidneys of volume depletion model mice was examined by immunostaining. DAPI (blue). Magnification: ×1000. (**E**) Urinary activin A level in normal mice, volume depletion (VD) model mice and I/R model mice (at 48 hr after reperfusion, 22 min ischemia). *p < 0.05 vs. control. ^#^p < 0.05 vs. IR.

To confirm that urinary activin A increases in renal AKI, we examined whether urinary activin A was detectable in mice with volume depletion that mimics pre-renal AKI. Volume depletion significantly induced body weight reduction (Fig. 6A) and increased BUN levels (Fig. 6B), but not serum creatinine levels (Fig. 6C). In contrast to the ischemic injury model, activin A was not present in tubular cells of the kidneys in volume depletion model mice (Fig. 6D). Urinary activin A was slightly detected in mice with volume depletion, which was significantly lower than that in mice with AKI (Fig. 6E).

## Discussion

In the present study, we demonstrated that urinary activin A was significantly increased in both ischemic mice (Fig. 3A) and AKI patients (Fig. 5A). There are several mechanisms by which activin A could appear in the urine in AKI. The first is that urinary activin A is derived from glomerular filtered activin A. The molecular weight of activin A is 25 kDa, a size that can be theoretically filtered by glomeruli. Similar to other urinary biomarkers, including NGAL^28^ or L-FABP^29^, glomerular filtered activin A might be reabsorbed by renal tubules through endocytosis in the normal kidney. Dysfunction of tubular reabsorption might lead to the presence of activin A in the urine. To support this idea, urinary activin A significantly increased in mice with renal ischemia at 3 hr after reperfusion, at which time βA subunit mRNA was not detected in the ischemic kidneys by *in situ* hybridization (Figs 1B and 3A). It is also possible that serum activin A was markedly elevated at 3 hr after reperfusion and the increased amount of glomerular filtered activin A causes urinary activin A. The second possible mechanism is that urinary activin A originates from tubular cells of the ischemic kidneys. Both βA subunit mRNA for activin A (Fig. 1B) and activin A protein (Fig. 1D) were present in tubular cells at 6 hr after reperfusion and thereafter. Since activin A-producing tubular cells are not proliferating (Fig. 2C) or apoptotic (Fig. 2D,E), the mechanism by which activin A expression is regulated is unknown. Nevertheless, these results suggest that activin A appears in the urine by tubular cell dysfunction or tubular damage in AKI.

Measurement of urinary activin A has several advantages for the early diagnosis and treatment of AKI in the clinical setting. We demonstrated here that activin A was present in proximal tubular cells of the ischemic kidneys, but not in normal kidneys (Fig. 1C). Activin A-positive area in ischemic kidneys increased according to the ischemic period (Fig. 4E). Urinary activin A level was positively correlated with the degree of activin A-positive area (Fig. 4H). On the other hand, renal ischemia for 15 min did not induce the elevation of serum creatinine level (Fig. 4C), but significantly increased urinary activin A (Fig. 4F). In one patient with drug-induced AKI, urinary activin A increased in parallel with serum creatinine level and rapidly decreased before the normalization of serum creatinine level (Fig. 5H). These data suggest that urinary activin A may serve as a sensitive biomarker reflecting the severity of tubular damage. Urinary activin A also appears to be valuable in distinguishing renal and pre-renal AKI. Similar to mice with AKI, urinary activin A was present in the urine of patients with renal AKI, but not in patients with pre-renal AKI (Fig. 5A). Although a slight increase in urinary activin A level was found in volume depletion model mice, its absolute value was significantly lower than that in the AKI mouse model (Fig. 6E). The expression of βA subunit for activin A was not detected in pre-renal AKI model mice by immunostaining (Fig. 6D). Collectively, urinary activin A might be a beneficial marker to distinguish between renal and pre-renal AKI according to its absolute values.

The transition of AKI to chronic kidney disease (CKD) has major clinical significance. Recent research has provided insights into the pathologic basis for failed recovery from AKI, such as tubular atrophy and renal fibrosis^30^. Late upregulation of NGAL and KIM-1 could be a useful marker for sustained renal injury after AKI^31^. Activin A acts as a potent inducer of renal fibrosis^32,33^, and the activin receptor type IIA ligand trap protects against vascular disease and renal fibrosis in mice with CKD^34^. Given that activin A inhibits tubular regeneration of the kidney after renal ischemia^25–27^, activin A produced by tubular cells inhibits tubular regeneration in a paracrine manner and induces incomplete recovery from AKI. In the present study, we could not clarify if the urinary activin A level reflects renal prognosis in AKI patients. Sustained activin A expression in the ischemic kidneys might be involved in the transition from AKI to CKD.

There are several limitations in this study. First, we have no data regarding urinary activin A in children with AKI. AKI in children is associated with increased mortality and prolonged hospital stay and may also be associated with long-term CKD development. It has been reported that urinary activin A in infants who developed intraventricular hemorrhage was significantly higher than in controls at all monitoring time-points^35^. Urinary activin A seems to be a promising tool for identifying preterm infants at risk of intraventricular hemorrhage. Previous studies also demonstrated that urinary activin A could be detected in the urine of pregnant females with pre-eclampsia^36^. Normal pregnancy urine samples had very low levels of activin A. In contrast, patients with pre-eclampsia had significantly higher levels of activin A compared to controls. Therefore, the advantage of urinary activin A is limited to patients except for infants and pregnant women. Although it was not statistically significant, urinary NAG potentially have strong negative correlation with urinary activin A (Fig. 5F). This unexpected data seems to be due to the difference in timing of urine collection from AKI patients. It is possible that urinary activin A increases, but urinary NAG does not, in patients with the early stage of AKI. In contrast, in patients with the later stage of AKI, it is expected that urinary NAG increases, but urinary activin A starts to decrease. To clarify if there is a significant correlation between urinary NAG and urinary activin in AKI, urine collected from equivalent stage of AKI patients should be used for analysis. We measured urinary activin A in AKI, but not in other kidney diseases. Given that urinary activin A was increased in lupus-prone MRL-lpr mice^37^, urinary activin A might be increased in patients with other kidney diseases including lupus nephritis. Further investigation will be needed to address this issue.

## Methods

### Experimental Protocols

Eight or twelve-week-old male C57BL/6j mice (Japan Charles River, Yokohama, Japan) were housed under specific pathogen-free conditions and provided with autoclaved food and sterile water ad libitum. Ischemia reperfusion injury and volume depletion were induced in mice as described in Supplemental data. All animal experiments were performed in accordance with Fundamental Guidelines for Proper Conduct of Animal Experiment and Related Activities in Academic Research Institutions and were approved by the Ethics Review Committee for Animal Experimentation of Gunma University (approval number 12-067).

### Renal Function and Urinary Protein

Serum or urinary creatinine and blood urea nitrogen (BUN) levels were assessed using a Hitachi 7180 autoanalyzer (Hitachi High-Technologies, Tokyo, Japan). Urinary protein was measured using a BCA assay kit (Pierce, Rockford, IL, USA).

### Immunohistochemical Analysis

Immunostaining was performed using a VECTASTAIN ABC-kit (Vector Laboratories) as described previously^25^. Briefly, paraffin-embedded sections (4 μm) were deparaffinized, hydrated according to standard methods, soaked in blocking serum, and incubated with primary antibody overnight at 4 °C. After washing with phosphate-buffered saline (PBS), sections were incubated with peroxidase-conjugated secondary antibody followed by diaminobenzidine and were counterstained with periodic acid-Schiff (PAS). Indirect fluorescent immunostaining was performed as follows^26^. Briefly, sections were incubated with fluorescein-labeled secondary antibodies (Alexa; Molecular Probes, Eugene, OR) and 4′,6′-diamidino-2′-phenylindole dihyrochloride (DAPI). Fluorescent images were recorded with the BZ-X700 all-in-one fluorescence microscope (KEYENSE, Osaka, Japan). For the immunostaining control, the primary antibody was replaced with PBS, which did not show positive staining, confirming specificity. Primary antibodies used in this study were shown in Supplemental data. Quantification of activin A-positive areas was performed by measurement of positive area in five randomly selected fields of the outer medulla at ×200 magnification using ImageJ software (National Institutes of Health, Bethesda, MD).

### Histological Examination

PAS-stained sections were microscopically examined at the indicated periods after reperfusion. The changes observed were limited to the outer medulla, where tubular damage is most obvious, and were graded as follows: 0, normal; 1, areas of tubular dilation, necrosis, hemorrhage, and cell desquamation involving <20% of the fields; 2, similar changes involving >20% but <40% of the fields; 3, similar changes involving >40% but <60% of the fields; 4, similar changes involving >60% of the fields. Five sections per mouse were used for analysis.

### Terminal Deoxynucleotidyl Transferase-Mediated dUTP-Nick-End-Labeling

For identification of nuclei with DNA strand breaks at the cellular level, the terminal deoxynucleotidyl transferase-mediated dUTP-nick-end-labeling (TUNEL) method was performed using an apoptosis *in situ* detection kit (Takara, Tokyo, Japan) according to the manufacturer’s instructions.

### Real-time PCR

Tissues were homogenized using a microhomogenizer and total RNA was extracted using RNAiso (Takara). First-strand cDNA was made from total RNA using SuperScript III First-strand (Invitrogen, Carlsbad, CA) according to the manufacturer’s instructions. Real-time PCR was performed using the ABI 7300 Real-time PCR System (Applied Biosystems, Foster City, CA) as shown in Supplemental data.

### ELISA

Urinary and serum human/mouse activin A, urinary human/mouse KIM-1 (R&D Systems), and urinary mouse NGAL (BIOPORTO, Hellerup, Denmark) was measured by ELISA according to the manufacturer’s instructions.

### *In Situ* Hybridization

*In situ* hybridization was performed using a InHyb *In Situ* Hybridization Kit (BioChain Institute Inc., Newark, CA) as described in Supplemental data.

### Patients

Patients who were admitted to Gunma University Hospital for the diagnosis and treatment of AKI (n = 18) were enrolled. Informed consent was obtained from all participants. All experiments were performed in accordance with relevant guidelines and regulations. This study was approved by the ethical committee on human research of Gunma University Graduate School of Medicine (approved number 855). Urine and serum were collected from patients with renal AKI (n = 13) (drug-induced 5, hypercalcemia 1, rhabdomyolysis 1, sepsis 1, granulomatosis with polyangitiis 1, HELLP syndrome 1, and cast nephropathy 3) and pre-renal AKI (n = 5) (dehydration) when the diagnosis was made or following recovery from AKI.

### Statistical analysis

Statistical analysis was performed using SPSS Statistics 24 (Chicago, IL). The significance of differences between means was compared using a t-test. When comparing the means of more than two variables, data were analyzed using Kruskal-Wallis test followed by the Mann-Whitney U test using Bonferroni correlation to adjust the probability. Correlation was analyzed with Spearman’s rank correlation test coefficients. P < 0.05 was considered significant.

## Electronic supplementary material

Supplemental data
