# Supplementary material for: Identification of Urinary Activin A as a Novel Biomarker Reflecting the Severity of Acute Kidney Injury
Source: Sci Rep. 2018 Mar 26;8:5176. doi: 10.1038/s41598-018-23564-3 (PMC5980079; doi:10.1038/s41598-018-23564-3)
Supplement: Supplementary file 1 — Supplemental data [file 41598_2018_23564_MOESM1_ESM.pdf]

Identification of Urinary Activin A as a Novel Biomarker Reflecting the Severity of Acute Kidney Injury

Shunsuke Takahashi, Masao Nakasatomi, Yoshinori Takei, Hidekazu Ikeuchi, Toru Sakairi, Yoriaki Kaneko, Keiju Hiromura, Yoshihisa Nojima, Akito Maeshima

Department of Nephrology and Rheumatology, Gunma University Graduate School of Medicine

Running Title: Urinary Activin A in Acute Kidney Injury

Key words: activin A, acute kidney injury, urinary biomarker

Address correspondence to: Akito Maeshima, M.D., Ph.D.

Department of Nephrology and Rheumatology,

Gunma University Graduate School of Medicine

3-39-15 Showa, Maebashi 371-8511, Japan

Phone: +81-27-220-8166; Fax: +81-27-220-8173

E-mail: amaesima@gunma-u.ac.jp

Supplemental Data

Ischemia reperfusion injury

Ischemia reperfusion injury was induced in mice as described below. Briefly, the mice were anesthetized with pentobarbital sodium (50 mg/kg) by intraperitoneal injection. Shortly thereafter, buprenorphine (50 μg/kg) was administered subcutaneously to control pain and distress. After deep anesthesia, the abdominal cavity was exposed via a midline incision. Renal ischemia was induced by clamping both renal arteries for 15, 22, 25 or 30 min using a non-traumatic vascular clamp. Core body temperature was maintained at 37°C by placing the animal on a homoeothermic table. After the ischemia periods indicated above, the clamps were removed and reperfusion of the kidneys was confirmed visually. The mice were subsequently sacrificed after various post-ischemic periods. The kidneys were removed and frozen for RNA extraction or fixed in 10% formalin for routine paraffin embedding and sectioning for histologic analysis. Urine was collected from individual mice housed in metabolic cages. After centrifugation at 10,000 rpm for 5 min, all urine samples were stored at -80°C until the day of analysis.

To mimic pre-renal AKI, simple volume depletion was induced in mice by water intake restriction. The degree of dehydration was evaluated by measurement of body weight recorded every 24 hr. Urine and blood samples were collected at 48 hr after initiation of water restriction and then mice were sacrificed.

Materials

Primary antibodies used in this study were as follows: rabbit anti-inhibin beta A antibody (ab97705) (Abcam, Cambridge, UK), mouse anti-neutrophil gelatinase-associated lipocalin (NGAL) antibody (AF1857), mouse anti-kidney injury molecule-1 (KIM-1) antibody (AF 1817) (R&D Systems, Minneapolis, MN), goat anti-aquaporin 2 antibody (sc-9882), goat anti-Tamm-Horsfall-protein antibody (sc-19554), mouse anti-E-cadherin antibody (sc-8426), goat anti-proliferating cell

nuclear antigen (PCNA) antibody (sc-9857) (Santa Cruz Biotechnology, Santa Cruz, CA), rabbit anti-cleaved caspase-3 antibody (#9661; Cell Signaling Technology, Danvers, MA). Fluorescein lotus tetragonolobus lectin (LTL) (FL-1321) was from VECTOR Laboratories (Burlingame, CA).

In Situ Hybridization

In situ hybridization was performed using a InHyb In Situ Hybridization Kit (BioChain Institute Inc., Newark, CA). Hybridization probes were obtained from Genostaff Co. Ltd (Tokyo, Japan). After deparaffinization and rehydration, sections were fixed in 4% paraformaldehyde in DEPC-PBS at room temperature for 20 min. After digestion with 10 μg/ml proteinase K at 37°C for 20 min, sections were postfixed in 4% paraformaldehyde in DEPC-PBS at room temperature for 15 min. Sections were incubated with pre-hybridization solution for 3 hr at 50°C. Hybridization was performed with sense or antisense probes (2.5 ng/μl) at 50°C for 16 hr. After hybridization, sections were washed once in 2 × SSC at 45°C for 10 min, 1.5 × SSC 45°C for 10 min, and washed twice in 0.2 × SSC at 37°C for 20 min. Sections were incubated in 1× blocking solution for 60 min at room temperature and then incubated in a 1:200 diluted solution of AP-conjugated anti-digoxingenin antibody for 1 hr, before washing and detection of the label with nitroblue tetrazolium chloride and 5-bromo-4-chloro-3-indolyphosphate.

Real-time PCR

Real-time PCR was performed using the ABI 7300 Real-time PCR System (Applied Biosystems, Foster City, CA). Reactions included 5 μl of a SYBR Green Real-time PCR Master Mix (TOYOBO, Osaka, Japan), 0.2 μl of 3' primer, 0.2 μl of 5' primer, and 1 μl of cDNA. Samples were incubated at 50°C for 2 min, then at 95°C for 1 min, followed by 35 cycles of 15 sec at 95°C, 15 sec at 62°C and 60 sec at 72°C. The expression of each gene was quantified in separate tubes with the following primers; mouse inhibin βA (143 bp) sense 5'-GATCATCACCTTTGCCGAGT-3',

antisense 5’-TGGTCCTGGTTCTGTTAGCC-3’; and mouse GAPDH (460 bp) sense, 5’-TGCTGAGTATGTCGTGGAGTCTA-3%’, antisense 5’-AGTGGGAGTTGCTGTTGAAATC-3’. Data was expressed as the relative expression normalized to GAPDH. Agarose gel electrophoresis of the PCR products after quantification by real-time PCR showed single bands of the expected size (data not shown).
